# Benefits of adding food education sessions to an exercise programme on cardiovascular risk factors in patients with type 2 diabetes

**DOI:** 10.1017/jns.2021.50

**Published:** 2021-08-11

**Authors:** Carlos Eduardo Gonçalves da Costa Vasconcelos, Maria Manuela Lobato Guimarães Ferreira Cabral, Elisabete Conceição Pereira Ramos, Romeu Duarte Carneiro Mendes

**Affiliations:** 1 University of Trás-os-Montes e Alto Douro, Quinta de Prados, 5000-801Vila Real, Portugal; 2 School of Education of Viseu, Polytechnic Institute of Viseu, Rua Dr. Maximiano Aragão 41, 3504-501 Viseu, Portugal; 3 EPIUnit – Instituto de Saúde Pública, Universidade do Porto, Rua das Taipas 135, 4050-091 Porto, Portugal; 4 Departamento de Ciências da Saúde Pública e Forenses e Educação Médica, Faculdade de Medicina, Universidade do Porto, Alameda Prof. Hernâni Monteiro, 4200-319 Porto, Portugal; 5 Northern Region Health Administration, Rua de Santa Catarina 1288, 4000-477Porto, Portugal

**Keywords:** Cardiovascular risk factors, Exercise programme, Food education sessions, Type 2 diabetes

## Abstract

To evaluate the impact of adding food education sessions to an exercise programme on cardiovascular risk factors in middle-aged and older patients with type 2 diabetes (T2D), a randomised parallel-group study was performed. Glycated haemoglobin, body mass index (BMI), waist circumference, fat mass (FM) and blood pressure were assessed at baseline and after 9 months. The recruitment was made in three primary healthcare centres from Vila Real, Portugal. Thirty-three patients (65⋅4 ± 5⋅9 years old) were engaged in a 9-month community-based lifestyle intervention programme: a supervised exercise programme (EX; *n* = 15; combined aerobic, resistance, agility/balance and flexibility exercise; three sessions per week; 75 min per session); or the same exercise programme plus concomitant food education sessions (EXFE; *n* = 18; 15-min lectures and dual-task strategies during exercise (answer nutrition questions while walking); 16 weeks). Significant differences between groups were identified in the evolution of BMI (*P* < 0.001, 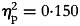) and FM (*P* < 0.001, 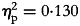), with best improvements observed in the EXFE group. The addition of a simple food education dietary intervention to an exercise programme improved body weight and composition, but not glycaemic control and blood pressure in middle-aged and older patients with T2D.

## Introduction

Type 2 diabetes (T2D) is a challenging public health issue, with adverse effects on health and economy^1^. Patients with T2D have a 15 % increased risk of all-cause mortality compared with people with normal glucose tolerance^2^. Cardiovascular diseases, more specifically stroke and coronary heart disease, are the main causes of death in individuals with T2D^3^, mostly for those with ≥65 years old^4^. Unhealthy dietary behaviours and physical inactivity, obesity, high blood pressure and hyperglycaemia are major cardiovascular risk factors associated with T2D^5^^,^^6^. It is well established that lifestyle modifications programmes incorporating dietary and/or exercise interventions improve cardiovascular risk factors in patients with T2D, when compared with a control group^7^^,^^8^.

In a meta-analysis conducted in T2D patients, combined aerobic and resistance exercise reduced significantly HbA1c by 0⋅67 %, WC by 23⋅1 cm and SBP by 3⋅59 mmHg^9^.

Combined exercise programmes also induced significant improvements on BMI^10^^,^^11^, FM^12^^,^^13^ and DBP^14^^,^^15^.

However, to the best of our knowledge, only one randomised controlled trial^16^, conducted in the United States of America during 14 weeks, has assessed if the addition of a nutritional intervention to an exercise programme leads to improvements in cardiovascular risk factors in middle-aged and older patients with T2D. In the present study, exercise intervention consisted of aerobic exercise, performed during 60 min, three to four times a week. Regarding nutritional intervention, a diet composed of 40 % fat, 40 % carbohydrates and 20 % protein was prescribed to the participants.

However, according to international physical activity recommendations for T2D control^17^^–^^19^, combined aerobic and resistance exercise programmes should be applied in these patients. In what concerns nutritional intervention, there is a lack of easy-to-implement strategies among community settings in order to develop the capacity for self-management of diet^20^. Previous findings from our research team showed that a food education programme improved the dietary pattern of T2D patients through significant changes in polyunsaturated fat intake and servings of vegetables per day^21^.

Thus, the present study aimed to evaluate if the addition of a simple food-education dietary intervention confers additional effect to a combined exercise programme on cardiovascular risk factors in middle-aged and older patients with T2D.

## Methods

### Study design

The present study was a randomised parallel-group trial conducted in the city of Vila Real, Portugal. Participants were recruited to a 9-month community-based lifestyle intervention programme for patients with T2D and engaged, following simple randomisation procedures, to an exercise programme only (EX) or to the same exercise programme combined with concomitant food education sessions (EXFE). All patients received information to maintain their diabetes management (lifestyle-related physical activity and pharmacological plan), and to continue with their medical consultations during the study.

Glycaemic control (glycated haemoglobin (HbA1c)), anthropometric profile (body mass index (BMI) and waist circumference (WC)), body composition (fat mass (FM)) and blood pressure (systolic blood pressure (SBP) and diastolic blood pressure (DBP)) were assessed before (baseline) and after the lifestyle intervention programme (9 months).

### Participants

The implementation of the community-based lifestyle intervention programme was planned for two groups of twenty participants each (the limit of our human, material and infrastructure resources), representing a total of 274 h of intervention (135 h in the EX group and 139 h in the EXFE group). Predicting an initial refusal rate of 25 %, primary healthcare medical doctors from three primary healthcare centres from Vila Real (Portugal) were asked to select sixty-six participants (twenty-two from each primary health centre) according to the following inclusion criteria: T2D diagnosed at least for 6 months; aged between 50 and 80 years old; non-smokers; not engaged in supervised exercise; independent living in the community; medical recommendation for lifestyle intervention; known medical history; diabetes comorbidities under control (diabetic foot, retinopathy and nephropathy); no cardiovascular, respiratory and musculoskeletal contraindications to exercise; without major changes in gait and balance; not started insulin therapy in the past 3 months.

Our research team received information of sixty-seven primary healthcare patients with T2D. Forty-two agreed to participate in the study and were randomised for the EX (nineteen patients) or EXFE (twenty-three patients) group.

Medical events with hospitalisation (diseases, accidents or surgeries) during the study period were determined as exclusion criteria from final analysis. The level of adherence was not considered exclusion criteria for final analysis. Dropouts are explained in the Results section.

The present study was conducted according to the guidelines laid down in the Declaration of Helsinki and all procedures involving patients were approved by the Health Committee of the Portuguese Northern Region Health Administration. Written informed consent was obtained from all patients.

### Exercise programme

Patients participated in ‘*Diabetes em Movimento*’, a community-based exercise programme for patients with T2D, developed in Portugal^22^^,^^23^ according to international physical activity recommendations for T2D control and falls prevention^17^^,^^24^.

This supervised exercise programme was implemented during 9 months and consisted of three exercise sessions per week, 75 min per session. Each session was performed at the municipal sports complex and organised according to the following structure: warm-up, aerobic exercise, resistance exercise, agility/balance exercise and flexibility exercise. The warm-up consisted of 10 min of brisk walking. In aerobic exercise, patients accomplished 30 min of continuous brisk walking. In resistance exercise, patients accomplished 20 min of muscle-strengthening through the performance of six exercises (three for the lower limbs and three for the upper limbs and torso; with chairs, gymnastic balls and dumbbells) in circuit mode: the number of circuits of the resistance exercises increased progressively from one (in the first month for an adaptation phase) to four (in the last 5 months); the number of repetitions in unilateral and bilateral exercises was respectively 30 (performed alternately) and 20; patients had the opportunity to increase the load (dumbbells weight) depending on local muscle fatigue. One agility/balance exercise was performed in each session (traditional games or small-sided games), during 10 min. In the last part of the session, patients performed 5 min of static (15 s in each position) and dynamic (10 repetitions) stretches, with the support of chairs.

To induce stimuli variability, five different exercise sessions were successively applied over time, each of them with different aerobic, resistance and agility/balance exercises. All exercise sessions were supervised by an exercise professional and a nurse, and were planned to have moderate intensity (12–13 points on Borg rating of perceived exertion scale with 6–20 points). In the end of each session, participants were asked to rate and register perceived exercise intensity. Participants’ attendance was also registered.

### Food education sessions

**Fig. 1.**
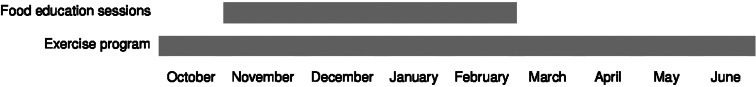
Food education sessions and exercise programme timeline.

Patients in the EXFE group received the same exercise intervention (‘*Diabetes em Movimento*’ exercise programme), as those in the EX group, plus concomitant food education sessions during 16 weeks (Fig. 1), based on American Diabetes Association (ADA) recommendations for dietary intake^25^, and International Diabetes Federation (IDF) nutrition teaching modules^26^. On each week, a different nutrition-related content (Table 1) was addressed by an exercise professional with professional qualification in the area of Nutrition through two sessions: (1) a theoretical session of 15 min performed through an interactive teaching method before one exercise session (instructing participants in a way they are actively involved with their learning process) and (2) dual-task strategies integrated in another exercise session (during aerobic exercise (brisk walking), patients had to interpret food labels or to give individual or group answers to nutritional questions through a traffic light system or multiple choice answer). Overall, guidance was given on how many portions of each food group to choose and participants were encouraged to eat foods with low glycaemic index and load, to avoid added sugars, to increase fruit, vegetables and soup consumption and to reduce fat, alcohol and salt intake. To favour changes in dietary habits, three behavioural change techniques were used: pros and cons, information about health consequences and instructions on how to strengthen behaviour change.

**Table 1.** Contents of food education sessions

Week	Contents
Week 1	Diabetes, insulin and glycaemia
Week 2	Functions of nutrients
Week 3	Sources of nutrients
Week 4	Food Wheel (fruit, vegetables, cereals, rice and potatoes)
Week 5	Food Wheel (meat, fish and eggs, dairy products, fats and oils)
Week 6	Glycaemic index and glycaemic load
Week 7	Added sugars
Week 8	Carbohydrate counting
Week 9	Food label interpretation (carbohydrates; sugars)
Week 10	Food label interpretation (fats; saturated fats)
Week 11	Dietetic products (lean, diet, light, zero)
Week 12	Fats
Week 13	Soup and salt
Week 14	Drinks
Week 15	Cooking methods
Week 16	Meal planning and the healthy eating plate

### Evaluations

Glycaemic control, through HbA1c, was assessed by a fasting (minimum of 8 h) venous blood analysis according to standard international laboratory methods. BMI was calculated by measuring body mass (kg) and height (m) (BMI = mass/height^2^) using a digital weight scale (SECA 778, SECA Corporation, Hamburg, Germany) with a stadiometer (SECA 220, SECA Corporation, Hamburg, Germany). WC was determined using an anthropometric tape at the umbilical reference (SECA 201, SECA Corporation, Hamburg, Germany). FM was assessed with bioelectrical impedance analysis (Tanita, BC-418 MA) after an overnight fast. Blood pressure (SBP and DBP) was measured with participants seated after a 10-min rest, by an automatic digital blood pressure device (BP-8800, Colin Corporation, Komaki, Japan), according to international recommendations^27^. Three blood pressure measurements were performed and the average was used as the measured value.

### Data analysis

Data are presented as mean ± standard deviation (sd) for continuous variables and as proportions (number and percentage) for categorical variables.

*χ*^2^ test was used to compare differences in proportions of HbA1c, BMI and WC within groups.

To compare the time × group interaction effects on HbA1c, BMI, FM, WC, SBP and DBP, an analysis of variance (ANOVA) with repeated measures was performed. Partial eta^2^ values 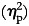 were reported to quantify the effect sizes. For all analyses, a level of statistical significance was defined as *P* < 0⋅05.

## Results

### Programme implementation

**Fig. 2.**
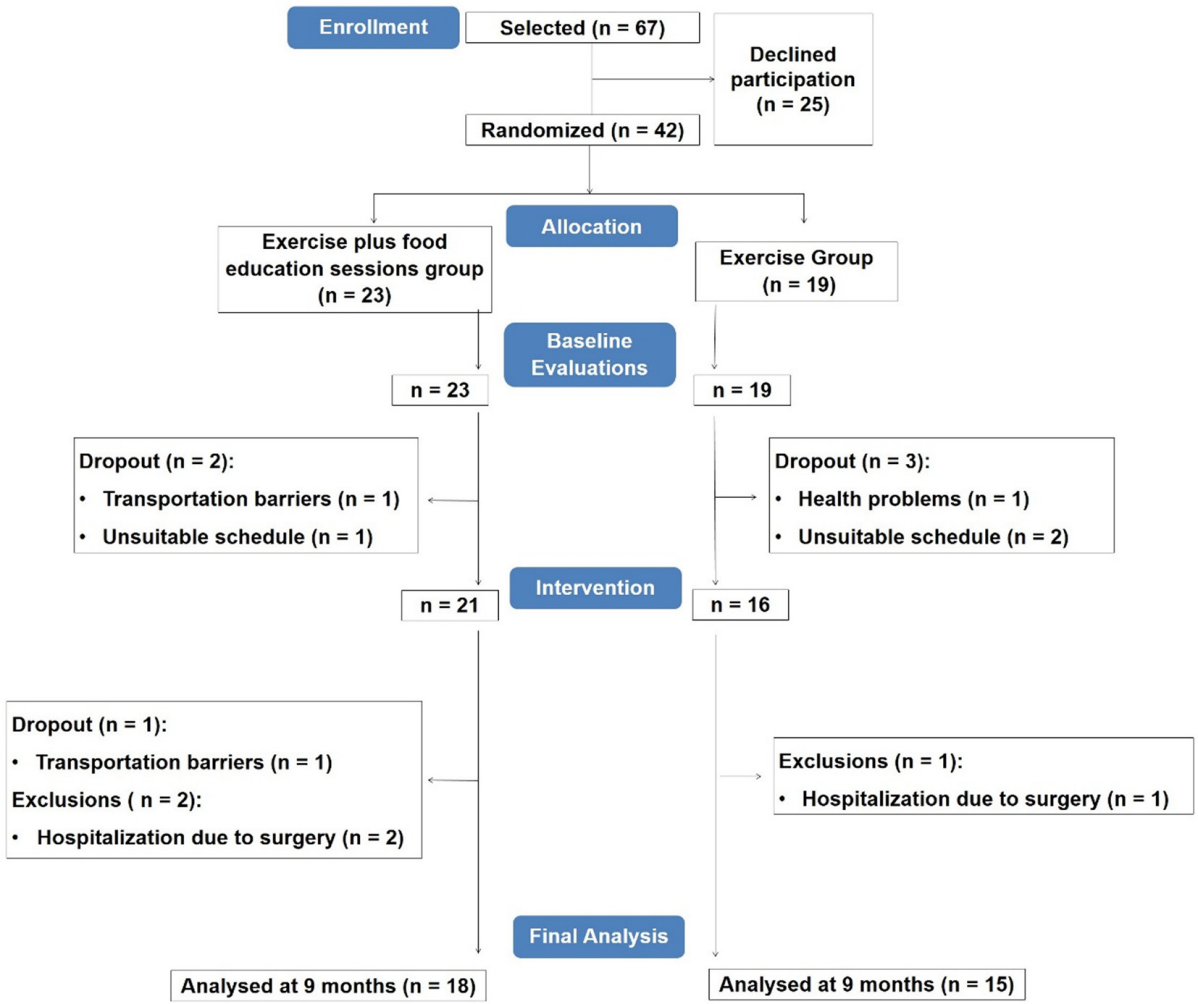
Participants’ flowchart.

From the individuals initially selected, 37 % refused to participate in the study, indicating transportation barriers or unsuitable schedule as reasons. Thus, forty-two participants were randomised and evaluated (twenty-three in the EXFE group and nineteen in the EX group). Prior to the start of the intervention, five participants dropped out (two in the EXFE group (unsuitable schedule, *n =* 1, transportation barriers, *n* = 1) and three in the EX group (health problems, *n =* 1, unsuitable schedule, *n =* 2)). Other dropout in the EXFE group was verified during the intervention due to transportation barriers (*n =* 1). Three patients were excluded from final analysis (two in the EXFE group and one in the EX group) because of hospitalisation due to surgery. In final analysis, eighteen patients from the EXFE group and fifteen patients from the EX group were included (Fig. 2).

Attendance to exercise sessions, expressed as the proportion of sessions attended, was 60⋅9 ± 25⋅0 % (ranging from 13⋅9 to 94⋅4 %) in the EXFE group and 52⋅9 ± 30⋅2 % (ranging from 3⋅7 to 98⋅2 %) in the EX group (*P* = 0⋅414). Attendance to food education sessions (EXFE group), expressed as the proportion of sessions attended, was 49⋅5 ± 27⋅2 % (ranging from 2⋅9 to 85⋅3 %).

### Participants’ characteristics

The mean age of the thirty-three participants (fourteen females) was 65⋅4 ± 5⋅9 years old. Participants had T2D diagnosed at 6⋅8 ± 5⋅2 years. Final sample characteristics are presented in Table 2.

**Table 2.** Characteristics of the study participants according to lifestyle intervention programme

Characteristics	EX Group (*n* 15)	EXFE Group (*n* 18)
Age, years (mean ± sd)	62⋅80 ± 5⋅52	67⋅61 ± 5⋅37
Gender, *n* (%)		
Male	10 (66⋅7)	9 (50⋅0)
Female	5 (33⋅3)	9 (50⋅0)
Educational level, *n* (%)		
≤4 years of school	9 (60⋅0)	11 (61⋅1)
5–9 years of school	3 (20⋅0)	5 (27⋅8)
>9 years of school	3 (20⋅0)	2 (11⋅1)
Personal net monthly income, *n* (%)		
<1000 €	2 (13⋅3)	10 (55⋅6)
1000–2000 €	7 (46⋅7)	6 (33⋅3)
>2000 €	6 (40⋅0)	2 (11⋅1)
Diabetes Duration, years (mean ± sd)	8⋅00 ± 5⋅72	5⋅72 ± 4⋅63
Medication, *n* (%)		
No medication	0 (0⋅0)	1 (5⋅6)
Oral antidiabetics	13 (86⋅7)	15 (83⋅3)
Oral antidiabetics + Insulin	2 (13⋅3)	1 (5⋅6)
Insulin	0 (0⋅0)	1 (5⋅6)
Hypertension, *n* (%)	15 (100⋅0)	18 (100⋅0)
Total Cholesterol, mg/dl (mean ± sd)	159⋅3 ± 24⋅3	184⋅8 ± 28⋅8
Triacylglycerols, mg/dl (mean ± sd)	123⋅2 ± 39	158⋅8 ± 94⋅9
Alcohol consumption^a^, *n* (%)	4 (27⋅0)	5 (28⋅0)

EX, Exercise; EXFE, Exercise plus food education.

a More than two standard alcoholic drinks per day.

### Outcomes

The mean values of HbA1c, BMI, WC, FM, SBP and DBP in the two evaluation moments in both groups are presented in Table 3.

**Table 3.** Mean values (±standard deviation) of glycaemic control and cardiovascular risk factors in the two evaluation moments in both groups

	EX group				EXFE group					
	Baseline	9 months	Δ	*p^a^*	Baseline	9 months	Δ	*p^a^*	*p^b^*	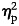
Glycated haemoglobin (%)	7⋅04 ± 1⋅11	7⋅01 ± 1⋅11	−0⋅03	0⋅811	7⋅16 ± 1⋅19	7⋅01 ± 1⋅29	−0⋅15	0⋅061	0⋅271	0⋅040
Body mass index (kg/m^2^)	30⋅84 ± 2⋅85	30⋅77 ± 3⋅21	−0⋅07	0⋅684	30⋅19 ± 2⋅81	29⋅52 ± 2⋅92	−0⋅67	0⋅001	0⋅026	0⋅150
Fat mass (kg)	29⋅02 ± 8⋅23	28⋅33 ± 8⋅76	−0⋅69	0⋅152	29⋅18 ± 7⋅54	27⋅19 ± 7⋅53	−1⋅99	0⋅000	0⋅039	0⋅130
Waist circumference (cm)	104⋅10 ± 6⋅57	103⋅77 ± 6⋅13	−0⋅33	0⋅601	104⋅04 ± 8⋅30	103⋅36 ± 7⋅54	−0⋅68	0⋅279	0⋅689	0⋅005
Systolic blood pressure (mmHg)	142⋅10 ± 14⋅86	133⋅62 ± 13⋅07	−8⋅48	0⋅061	141⋅45 ± 18⋅99	134⋅20 ± 18⋅42	−7⋅25	0⋅005	0⋅782	0⋅003
Diastolic blood pressure (mmHg)	85⋅18 ± 6⋅13	82⋅26 ± 7⋅39	−2⋅92	0⋅063	84⋅75 ± 9⋅87	84⋅04 ± 10⋅49	−0⋅71	0⋅631	0⋅293	0⋅039

EX, exercise; EXFE, exercise plus food education; Δ, variation between baseline and 9 months; *p^a^*, level of significance of the within-group differences; *p^b^*, level of significance of the time * group interaction effect; 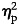, partial eta squared of the time * group interaction effect.

In the EX group, no significant differences were found in each one of the study variables (HbA1c, BMI, FM, WC, SBP and DBP) between the baseline evaluation and 9-month evaluation. The within-group comparison for EXFE group showed significant differences between the baseline evaluation and 9-month evaluation for BMI (*P* = 0⋅001), FM (*P* = 0⋅000) and SBP (*P* = 0⋅005). For HbA1c, WC and DBP, no significant differences were observed.

A significant time * group interaction effect was identified for BMI and FM, with better improvements in the EXFE group. For HbA1c, WC, SBP and DBP, no significant differences were observed between groups.

In Table 4, it was presented complementary data from cardiovascular risk factors in the two evaluation moments in both groups.

**Table 4.** Complementary data from cardiovascular risk factors in the two evaluation moments in both groups

	EX group (*n* 15)				EXFE group (*n* 18)			
	Baseline	9 months	Δ (%)	*p*	Baseline	9 months	Δ (%)	*p*
Glycated haemoglobin (>6⋅5 %), *n* (%)	8 (53⋅3)	8 (53⋅3)	0⋅0	0⋅837	12 (66⋅6)	10 (55⋅6)	−10⋅0	0⋅494
Body mass index (>30 kg/m^2^), *n* (%)	10 (66⋅6)	9 (60⋅0)	−6⋅6	0⋅705	9 (50⋅0)	7 (38⋅9)	−11⋅1	0⋅502
Abdominal obesity (cm), *n* (%)								
Men (≥94 cm)	9 (60⋅0)	9 (60⋅0)	0⋅0	1⋅000	9 (50⋅0)	9 (50⋅0)	0⋅0	1⋅000
Women (≥80 cm)	5 (33⋅3)	5 (33⋅3)	0⋅0	1⋅000	9 (50⋅0)	9 (50⋅0)	0⋅0	1⋅000

EX, exercise; EXFE, exercise plus food education; Δ, variation between baseline and 9 months; *p*, level of significance of the proportions’ comparison using *χ*^2^ test.

The proportion of participants that had glycated haemoglobin >6⋅5 % before and after the 9-month intervention remains the same in the EX group (*P* = 0⋅837). In the EXFE group, it was noticed a decrease of 10 % despite non-significant results (*P* = 0⋅494).

Compared with baseline values, the proportion of participants that had BMI >30 kg/m^2^ at 9 months decreased both in the EX and EXFE groups, with a higher reduction in the EXFE group (11⋅1 %; (*P* = 0⋅502) *v*. 6⋅6 %; (*P* = 0⋅705)).

The proportion of men and women that had abdominal obesity >94 and >80 cm before and after 9-month intervention remains the same both in the EX and EXFE groups (*P* = 1⋅000).

## Discussion

Food education sessions added benefits to an exercise programme in the control of BMI and FM. Diet and physical activity are two lifestyle factors that contribute to the prevention of cardiovascular diseases in individuals with T2D^28^ and the results of the present study show the value of combining both.

The efficacy of the exercise programme used in our trial (‘*Diabetes em Movimento*’) in cardiovascular risk factors in patients with T2D has been already demonstrated by Mendes *et al.*^22^. However, it must be stressed that regarding exercise programme attendance, the study of Mendes *et al.*^22^ used a per-protocol analysis in which attendance to the exercise sessions <65 % was considered an exclusion criteria, which led to higher mean exercise attendance values compared with our trial (80⋅17 ± 10⋅28 % *v*. 57⋅24 ± 27⋅34 %). In this regard, our study exhibits very heterogeneous values, with exercise attendance ranging from 3⋅7 to 98⋅2 %. The effects of exercise on cardiovascular disease and mortality risk seem to follow a linear dose-response relation^29^. Moreover, contrary to our trial, in the Mendes *et al.* study^22^, T2D patients performed a pre-participation treadmill stress test, which allowed the performance of vigorous-intensity exercise during the exercise programme (13⋅5 ± 1⋅4 points *v*. our study with 12⋅7 ± 0⋅8 points). Higher exercise intensities result in greater acute and chronic glycaemic control in T2D patients^30^^–^^32^. The fact that this exercise programme includes both aerobic and resistance exercise seems crucial for inducing benefits since isolated aerobic or resistance exercise effects on HbA1c are less pronounced than combined aerobic and resistance exercise^33^. A review of exercise prescription guidelines for patients with T2D^17^ pointed that, for the control of T2D and related cardiovascular risk, there is an international consensus for a combination of at least 150 min of moderate-to-vigorous-intensity aerobic exercise (minimum 3 d per week) and resistance exercise for major muscle groups (spread over at least 2 d a week). It is also recommended flexibility exercise as a complement of aerobic and resistance exercise. Besides, as the mean age of our sample was 65⋅4 ± 5⋅9 years old, these patients should perform agility/balance exercises to improve postural stability and reduce the risk of falls^24^^,^^34^.

Regarding our dietary intervention, although individualised nutrition therapy was recommended for T2D patients^25^, logistic constraints related to a community-based lifestyle intervention programme made us choose food education sessions. These sessions were based on recommendations from ADA and IDF^25^^,^^26^. As lectures are considered the most effective strategy to transmit declarative knowledge^35^ and attention declines with age^36^, one of the teaching methods used in our study was short duration lectures (15 min each). Besides, dual-task problem-solving strategies were used to allow the reinforcement of the contents in the exercise sessions. This innovative method is also essential in the prevention of falls^37^ and to prepare elderly for the dual-task activities of the daily life.

HbA1c is the gold standard indicator of long-term glycaemic control^38^. In line with the study of Giannopoulou *et al.*^16^, our dietary intervention had no additional significant benefit to the exercise programme in HbA1c levels, although the EXFE group observed a higher decreased HbA1c levels. One possible reason for the non-significant results of our study was the fact that HbA1c baseline levels were near 7 %, making it more difficult to lower these values.

Obesity is strongly related to higher risk of cardiovascular disease incidence and mortality^39^. According to World Health Organization^40^ that classifies those with BMI ≥ 30 kg/m^2^ as obese, 57⋅5 % of our patients were obese at baseline evaluation. Behavioural modifications, such as the promotion of healthy eating and regular physical activity, are the core of obesity management^41^. Although a meta-analysis of Norris *et al.*^42^ concluded that individuals with T2D had difficulties losing weight, in our trial the addition of food education sessions to an exercise programme succeeded in the decrease of BMI. The BMI is only a measure of weight relative to height and does not present information about FM^43^. According to Zeng *et al.*^44^, body FM is a better predictor of cardiovascular risk factors than BMI. It is common that patients with T2D had low basal fat oxidation and increased lipogenesis rates^45^, which makes it difficult to lose fat. Nevertheless, our EXFE group significantly reduced FM when compared with the EX group. However, it is not only the total amount of fat, but also its location, that is relevant for cardiovascular disease^46^. In this regard, abdominal obesity has a contribution on cardiovascular disease that is independent of BMI^47^. WC values provide a measure of both intra-abdominal and subcutaneous abdominal adipose tissue^48^. Body fat distribution is strongly determined by genetic factors^49^. Effectively, several studies demonstrated that people do not lose fat in predetermined parts of the body^50^^,^^51^, which may justify the non-significant results between groups in WC in our study. The study of Giannopoulou *et al.*^16^ showed no advantage of adding a dietary intervention to an exercise programme in the three outcomes related to body composition (BMI, FM and WC).

Hypertension, defined as SBP ≥ 140 mmHg and/or DBP ≥ 90 mmHg, is a high prevalent cardiovascular disease risk factor^52^. The benefits of lowering blood pressure (BP) are related to the decrease of left ventricular mass and wall thickness, the reduction of arterial stiffness and improvement of endothelial function^53^. The main lifestyles interventions that are recommended to reduce BP is exercise, especially aerobic exercise^54^, and the Dietary Approaches to Stop Hypertension diet^53^. Our exercise programme matched with recommendations from American College of Sports Medicine for the management of hypertension^55^. Regarding the dietary intervention, there was no prescription of Dietary Approaches to Stop Hypertension diet, as it was based on food education sessions and our main goal was not just focused on the reduction of blood pressure. This last evidence potentially explains no additional benefit of our food education sessions to the exercise programme either on SBP or DBP. There are other studies that tested the same research design – the addition of nutrition education sessions to an exercise programme, although not in patients with T2D. These studies^56^^–^^60^ concluded that the addition of nutrition education sessions to an exercise programme in obese patients have led to significant improvements on weight loss, corroborating the results of our study.

The main limitation of the present study is related to its sample size. We were only able to enrol 25 % of the total number of patients needed according to sample size calculations. Insufficient sample size can explain the non-significant results in glycated haemoglobin and other endpoints, besides anthropometry and body composition.

According to Miller *et al.*^61^, recruitment of participants into long-term community-based lifestyle interventions, particularly adults with a chronic disease, is often challenging. In our trial, we encountered this difficulty plus some dropouts and exclusions that did not allow a larger number of patients in final analysis neither a subgroup analysis. The level of attendance to lifestyle programmes was other problem faced by our team. Besides the low average attendance values of the exercise sessions, as discussed previously, food education sessions also had low average attendance values (49⋅5 ± 27⋅2 %, ranging from 2⋅9 to 85⋅3 %), which can, in part, explain the absence of additional benefits in HbA1c, WC and BP.

The nature of our dietary intervention (short education sessions that can probably be delivered even by non-specialized medical and other healthcare specialities) makes it feasible and realistic in many healthcare settings.

To the best of our knowledge, this is the first randomised parallel-group study that evaluates the impact of adding food education sessions to an exercise programme on cardiovascular risk factors in European patients with T2D. Moreover, 60 % of our patients were at least 65 years old. This is of crucial importance as, if not controlled, diabetes will lead to 70 % of deaths from cardiovascular disease in individuals of this age^4^.

The present study highlights the importance of community-based lifestyle interventions as a complement of management of T2D in primary health care^62^. There is an urgent need for the implementation of this type of programmes combining exercise and food education sessions, and the application of effective strategies to increase patients’ motivation and attendance.

The addition of simple food-education dietary intervention to an exercise programme induced additional benefits on one cardiovascular risk factor, more specifically obesity, in middle-aged and older patients with T2D. The present study provides further support for the implementation of lifestyle intervention programmes for T2D patients in community settings combining exercise and nutrition education.
